# A Successful Case of Direct Transfusion for Pernicious Anæmia

**Published:** 1881-01

**Authors:** Charles Cary


### A SUCCESSFUL CASE OF DIRECT TRANSFUSION FOR PERNICIOUS ANÆMIA.

BY CHARLES CARY, M. D.

THE case I here report is one which was examined by some of the leading medical men of our city, and attracted no small amount of professional interest.

For those who were not present at the transfusion, and also for a number who did not follow the case to its termination, I report it, feeling sure that the practical points brought forward can prove of great service to those in the profession who wish to practice transfusion with little or no preparation, and the assurance of success in cases where death would occur purely for want of quality or quantity of blood.

The patient was admitted to the medical ward of the General Hospital, July 17, 1880, with the following sparse history: Born in the United States; residence, Buffalo; widowed, but had been separated from her husband for some years; a milliner by occupation, and aged 22. She dated the beginning of her illness from February last (five months), when she took cold at the menstrual period and has had suppression since. The rotundity of the body and general firmness of tissues were natural; *Anæmia* very marked; had dizziness whenever in the erect position and stated that she had frequent epistaxis amounting by measurement at one time to a pint.

A syphilitis history cannot be established.

Ordered temperature and pulse record; and the patient to be kept under observation.

July 18th. The following points have been discovered in the last twenty-four hours:

Patient had slight chilliness in the morning, followed by a rise of temperature (102°) in the evening, also has vaginal discharge.

Speculum shows muco-purulent discharge from os uteri; uterus of normal measurement, vaginal mucus membrane injected. External palpation gives great pain in left iliac fossa on deep pressure. Ordered anodyne cotton applied to presenting portion of uterus, night and morning; quinine grs. v and tinc. iron m. x. three times a day. Tincture of iodine applied freely night and morning over iliac region.

July 24. Patient more anæmic; leucorrhœa ceased.

Dr. James P. White was called in consultation and could not discover uterine disease sufficient to account for such extreme anæmia; vaginal mucus membrane much improved. Temperature remains about the same, viz: 99 in A. M., 101 P. M., sometimes reaching 102°; pulse generally about 110; tongue clean.

Dr. White advised the continuance of iron and quinine, and suggested codeia gr. i, as often as necessary to maintain perfect quiet.

July 26. Patient comfortable, eats abundantly, has no impaired digestion, but is becoming weaker and more anæmic; pain continues in left iliac region but has abated somewhat. Ordered to discontinue Tr. iron, and give ferrum redactum grs. iii. Gave beef extract, milk and whiskey in abundance.

July 28. Increased reduced iron to grs. iv.

July 30. Ordered quinine to be discontinued for the day, and grs. xx to be given in the space of one hour on the day following. Pain in iliac region diminished. Bowels were moved by enema, which up to the present time had been ordered every second or third day.

July 21. Dr. Thos. F. Rochester called in consultation; patient failing, though nourishment is taken in *immense* quantities. No new developments. Ordered dialyzed iron m. xx. Fl. Ext. Ergot m. xx, each alternately three times a day, to be administered hypodermically.

Aug. 1. Sphygmographic tracings taken and continued from this time on through sickness. Hypodermic remedies as ordered above cannot be tolerated, and are discontinued.

There are at this time unmistakeable signs of an approaching fatal termination. Pulse 117, and barely appreciable; temperature 101°; involuntary micturition and marked cerebral failure; articulation slow and labored, with loss of memory.

On Aug. 2 a general consultation of staff was called to determine upon the advisability of transfusion. A variety of opinion was expressed as to the cause of the anæmia, but all concurred in the belief that death was imminent, and transfusion offered the only hope for her relief. The patient at this time was absolutely without color, not even a minute vessel could be detected in the conjunctiva; the gums were perfectly bleached, the lips white, and the body, though still round and firm, white as alabaster.

Aug 3. Transfusion performed in the presence of Drs. J. P. White, Thos. F. Rochester, E. M. Moore, Jr., Diehl, Hauenstein, Gay, Harrington, Hopkins, Wyckoff, Mynter, Loomis, Abbott and others.

The operation was conducted in the following manner: The patient's left median basilic vein was exposed by dissection, and Dr. Moore's transfusion canula was inserted, and allowed to remain, being held in place by rubber bands passed around the arm. The donor's (Dr. C. C. Fredericks) left median basilic vein was then exposed and raised, a band having been placed around the arm to produce venous engorgement. Up to this point, matters were conducted slowly for there was not a drop of blood drawn and no special pain was experienced by the patient.

Everything now being in readiness Dieulafoy's aspirator was used, being submerged in water to prevent the ingress of air, which was perhaps an unnecessary precaution. An incision was then made in the vein of the donor, and a blunt canula the full size of the vein introduced; the aspirator was thus readily filled with rich blood; its action was then reversed, but the tube from it was not connected with the canula in the recipient's arm until blood flowed from it; immediately upon the appearance of blood at the distal end of the tube, it was connected with the canula already in situ and the blood injected into the recipient's veins. The quantity thus introduced was ℥ ij.

The immediate effect upon the patient of this small quantity of blood was most surprising, she felt a warm sensation, a tremor passed over her, she gave a slight convulsive movement, her face flushed perceptibly and the operation was completed, occupying twenty seconds from the time the donor's vein was opened to the withdrawing of the bayonet trocar of Moore's canula.

Two hours after the operation the patient experienced what generally follows the transfusion of blood after prolonged exsanguinity, viz: a chill followed by a fever which reached 103½°.

Aug. 4. Patient much better, has had no involuntary micturition since yesterday, appears brighter and expresses herself as feeling much stronger.

From this time a rapid improvement took place, at first one vessel appeared on the conjunctiva, later it was joined by others until its color was normal, her lips red, and later her hands and nails showed a normal quantity of blood. She was much troubled for a time by a deep seated and violent pain in the hepatic region, the exact nature of which was not determined.

On Aug. 25th she was up and apparently well, though still feeble.

I have intended that this report should be a brief one, stating the facts as they existed and impressed me; the case was watched very closely and a most complete record of every detail preserved; I have abbreviated considerably, and cut those portions of the general record which seemed to have but little bearing upon the general course of the disease. It will be observed that there was great paucity of symptoms, the only two which constantly prevailed being, a progressive anæmia and a variation of morning and evening temperature.

I now wish to make one more statement which is of the greatest importance, and I shall forbear making any comment, leaving the profession at large the right to draw their own conclusions from what I state.

On Aug. 28, after the patient had been up and was nearly in a fit condition to be discharged, three weeks and four days after the transfusion, she was seized with general malaise, and the prodromic symptoms of typhoid fever followed in rapid succession, the fever itself developing shortly afterwards; on the same day Dr. C. C. Fredericks (the donor) developed the same disease, the two cases being about alike in grade.

The morning temperature running at about 102½, the evening 104, with a few exceptional days when it reached 104½, and upon two occasions attained the height of 105.

The fever left them both after a duration of about thirty days. The anæmia even during this run of fever did not re-appear.

